# Dietary fiber intake and metabolic syndrome in postmenopausal African American women with obesity

**DOI:** 10.1371/journal.pone.0273911

**Published:** 2022-09-02

**Authors:** Krista Lepping, Lucile L. Adams-Campbell, Jennifer Hicks, Mary Mills, Chiranjeev Dash

**Affiliations:** Georgetown Lombardi Comprehensive Cancer Center, Georgetown University Medical Center, Washington, D.C., United States of America; Endocrinology and Metabolism Population Sciences Institute, Tehran University of Medical Sciences, ISLAMIC REPUBLIC OF IRAN

## Abstract

Fiber intake may be associated with lower risk of metabolic syndrome (MetS) but data from metabolically unhealthy African American women is sparse. We examined the association of dietary fiber intake and MetS among postmenopausal African American women with obesity. Baseline cross-sectional data from the Focused Intervention on Exercise to Reduce CancEr (FIERCE) trial of 213 women (mean age 58.3 years) were used. Dietary intake was assessed by Food Frequency Questionnaires (FFQs). Multivariate linear and logistic regressions were performed to estimate associations of MetS with fiber intake and adherence to dietary fiber intake guidelines, respectively. Mean daily fiber intake was (10.33 g/1000kcal) in women with impaired metabolic health. We observed an inverse association of total fiber intake with MetS. One unit increase in energy-adjusted fiber intake was associated with a 0.10 unit decrease in the MetS z-score (p = 0.02). Similar results were obtained for both soluble and insoluble fiber. In multivariate-adjusted analyses, participants not adherent to fiber intake recommendations were more likely to have MetS as compared to those reporting intakes in the recommended range (adjusted odds ratio 4.24, 95% CI: 1.75, 10.30). Of the MetS components, high fasting glucose and high triglycerides were all associated with lower intake of fiber. Study participants who consumed a higher amount of fiber had a better overall metabolic profile and were less likely to have MetS in our cross-sectional analysis of postmenopausal African American women with obesity and unhealthy metabolic profiles.

## Introduction

Metabolic Syndrome (MetS), comprised of a combination of high blood pressure, elevated triglycerides, low levels of high-density lipoprotein (HDL), elevated blood glucose, and abdominal obesity, leads to a greater risk of cardiovascular disease, type 2 diabetes, and is established as an independent risk factor for breast cancer [1, 2]. Based on the 2007–2012 National Health and Nutrition Examination Survey (NHANES) data, the prevalence of MetS in US adults is 34.2% [3]. African American women are particularly affected by both MetS and its individual components. Overall, 36.7% of African American women in NHANES have MetS, and more specifically, 52.7% of African American women ages 50–69 years [3]. Additionally, African American women have higher prevalence of hypertension and abdominal obesity (38.5% and 48.6%, respectively) compared to their non-Hispanic white counterparts (31.1% and 35.9%, respectively) [3].

Underlying biological mechanisms of MetS are primarily obesity, insulin resistance, an inhibition of lipolysis, and an excess in fatty acids [4]. Obesity increases the mass of adipocytes, leading to changes in lipoproteins and their metabolization [5]. These alterations lead to an increase in the catabolism of HDL cholesterol, insulin resistance, and an increase in circulating fatty acids [5]. Poor diet/nutrition plays a significant role in these processes [6]. Studies suggest that high intake of meat and fried foods, consistent with a Western diet, leads to a higher incidence of MetS [7]. The adverse biologic effect of an unhealthy diet on MetS development is mediated through micro- and macronutrients. For example, meat, which is high in saturated fat, is associated with hypercholesterolemia, hypertension, and obesity [7]. In contrast, fiber intake is essential to maintaining good metabolic health [8].

Dietary fibers, categorized as soluble or insoluble, are a group of carbohydrates and lignin that cannot be digested or absorbed in the human body [9]. Soluble fiber, found in foods such as carrots, broccoli, and onions, is more easily fermented by colonic bacteria and has the ability to absorb water, ultimately leading to increases in food transit time, decreases in nutrient absorption, and the slowing of digestion [9]. Insoluble fiber, found in foods such as whole grain, nuts, and seeds, has the opposite effect of soluble fiber by decreasing food transit time in order to help relieve constipation [9]. Almost all plant sources of fiber contain both kinds, but the proportion varies from item to item. Fruits and vegetables that have high dietary fiber content can improve both insulin sensitivity and glucose control [10]. In addition, fiber may lead to reductions in blood pressure and LDL without negatively affecting HDL concentrations [11]. The Institute of Medicine’s daily recommendation of fiber for women is at least 21 grams per day or 14 grams per 1000 kcal [12]. Prior dietary surveys suggest that African American women have one of the lowest dietary fiber intake [13].

Previous studies have shown that low fiber intake among African American women impacts weight maintenance, contributing to an increased risk of obesity [13]. However, research on the association of dietary fiber intake and MetS is sparse in African Americans, particularly in postmenopausal African American women. In addition, it is unclear whether there is variability in fiber intake among women with obesity and whether this variability might be associated with MetS.

The aim of our study is to examine the relationship between dietary fiber intake and MetS among postmenopausal African American women with obesity in the Focused Intervention on Exercise to Reduce CancEr (FIERCE) trial. We hypothesize that, even in a relatively metabolically unhealthy population, participants with higher dietary fiber intake at baseline have a lower prevalence of MetS than those with lower dietary fiber intake.

## Materials and methods

### Design and participants

The FIERCE trial is a randomized controlled trial examining the effect of two exercise interventions on MetS and its components in postmenopausal African American women at high risk for breast cancer [14]. This analysis includes data from baseline observations in the FIERCE trial prior to the start of the intervention. The sample included 213 participants between the ages of 45 and 67, who were recruited from predominantly African American communities within the Washington, DC metropolitan area [15]. All participants were metabolically unhealthy with abdominal obesity and at least one additional MetS component at baseline. Exclusion criteria included being premenopausal; having any prior history of cancer, except non-melanoma skin cancer; having diabetes or using anti-diabetic medications (including insulin); currently exercising regularly (at least 20 minutes of moderate or vigorous activity for at least two times per week); currently enrolled in another physical activity and/or dietary study; and unable to commit to the FIERCE intervention schedule [15]. Written informed consent was obtained from all participants. This analysis includes data on all consented participants who completed baseline assessments. The study protocol was reviewed and approved by the Georgetown University–MedStar Health Institutional Review Board system.

### Metabolic variables

A diagnosis of MetS is defined as three or more of the following: systolic blood pressure ≥130 mm Hg or diastolic blood pressure ≥85mm Hg; HDL cholesterol <50; fasting glucose ≥100 mg/dL; triglycerides ≥150 mg/dL; and waist circumference ≥88cm [16]. Components of MetS were measured according to the US National Cholesterol Education Program Adult Treatment Panel III (ATPIII) using the following: systolic and diastolic blood pressure were measured using the average pressures after 10 and 20 minutes of rest in a seated position; fasting glucose, triglycerides, and HDL cholesterol were measured from collected venous blood samples that were drawn in the morning; and waist circumference was measured in centimeters using a measuring tape around the level of the navel [15]. Each component was dichotomized into the following factors using the ATPIII criteria: high systolic blood pressure, high diastolic blood pressure, high fasting glucose, high triglycerides, low HDL, and high waist circumference.

MetS was dichotomized for those who have three or more of the above factors and those who have less than three. A continuous MetS z-score was also calculated based on ATPIII criteria and the standard deviations for each MetS component from all participants in the FIERCE trial at baseline (n = 213). The following equation was used to calculate the MetS z-score: z-score = [(waist circumference– 88) / 13.72] + [(fasting blood glucose– 100) / 13.04] + [(mean arterial pressure– 100) / 11.26] + [(triglycerides– 150) / 61.17] + [(50 –HDL) / 18.22] [13]. Mean arterial pressure was calculated as [{(systolic blood pressure) + (diastolic blood pressure * 2)} / 3] [13].

### Dietary fiber

Dietary fiber was measured using the Block 2005 Food Frequency Questionnaire (FFQ), which includes 110 food items that are commonly consumed by African Americans and assesses usual dietary intake and patterns [15, 17]. The amount of total fiber, soluble fiber, and insoluble fiber ingested daily were collected, and daily intakes were classified as absolute intake (grams/day) as well as nutrient density (gram per 1000 kcal/day).

Additionally, total daily fiber intake among study participants was dichotomized into two additional variables based on adherence to dietary recommendations for absolute fiber intake (≥21 grams/day) and energy-adjusted fiber intake (14g/1000 kcal per day) [18].

### Covariates

Demographic and lifestyle variables were collected through self-reported questionnaires and used as covariates in analyses. These included age, smoking habits, education, and marital status. Anthropometric measures such as height and weight were collected and calculated into body mass index (BMI) [14]. Additionally, 7-day physical activity data was collected using the International Physical Activity Questionnaire (IPAQ) [19]. For each activity in the questionnaire, a metabolic equivalent task (MET) score was assigned based on its energy cost and multiplied by the amount of time spent in hours per week [14]. This total MET-hours/week represented the total amount of physical activity expended for each participant. The last variable used as a covariate was total energy intake, reported in kcal/day, as measured by the Block 2005 FFQ [17].

### Statistical analyses

Frequencies and descriptive statistics were used to describe the overall metabolic, dietary, and covariate variables among the total population, as well as stratified by those who were and were not meeting the daily fiber recommendations. Independent sample t-tests and chi-square tests were conducted to compare mean differences between these groups. The association of MetS and MetS components with adherence with fiber intake recommendations were analyzed by t-tests comparing means (SD) of MetS Z-score and MetS components across the 2 adherence groups. Multivariable logistic regression was used to test the association of MetS with adherence to fiber intake guidelines, and multivariable linear regression was used to model the association between fiber intake and the MetS z-score. Separate models were fit for total, soluble, and insoluble fiber intake. All multivariable analyses were adjusted for age, as well as for BMI, smoking, education, marital status, total physical activity, and total energy intake. All analyses were conducted using IBM SPSS statistical software version 28.0.

## Results

The study population characteristics by adherence to recommended daily fiber intake (categorized both as absolute intake and intake per 1000 kcals) are shown in Table 1. A majority of the participants did not meet the recommended fiber intake guidelines of 14g/1000 kcals (84%) and 21g/day (70%). As expected, higher absolute fiber intake was associated with higher total energy intake. However, after adjustment for energy intake participants not meeting the daily fiber density recommendations were more likely to have higher total energy intake and MetS prevalence than those meeting daily fiber intake recommendations.

**Table 1:** Baseline characteristics and metabolic syndrome by fiber intake guidelines.

Characteristic	Total Population (N = 213)	Fiber Intake ≥14g/1000 kcal per day (n = 34)	Fiber Intake <14 g/1000 kcal per day (n = 179)	Between Group P-Values	Fiber Intake ≥21 g/day (n = 62)	Fiber Intake <21 g/day (n = 151)	Between Group P-Values
	Mean (SD), %	Mean (SD), %	Mean (SD), %		Mean (SD), %	Mean (SD), %	
*Age (years)*	58.28 (5.003)	58.34 (4.73)	58.27 (5.06)	0.94	57.71 (4.86)	58.52 (5.06)	0.29
*BMI (kg/m* ^*2*^ *)*	35.84 (7.13)	34.82 (8.51)	36.04 (6.84)	0.36	35.04 (6.81)	36.17 (7.25)	0.29
*Smoking habit*				0.11			0.02*
Current	13.6%	2.9%	15.6%		24.2%	9.3%	
Former	33.3%	32.4%	33.5%		30.6%	34.4%	
Never	53.1%	64.7%	50.8%		45.2%	56.3%	
*Education*				0.15			0.12
≤ High School	7.5%	8.8%	7.3%		12.9%	5.3%	
High School/Some College	51.9%	35.3%	54.7%		53.2%	51.0%	
≥ College	40.6%	52.9%	38.0%		33.9%	43.0%	
*Marital Status*				0.15			0.78
Single/Never Married	29.6%	17.6%	31.8%		27.4%	30.5%	
Married/Living with Partner	25.8%	23.5%	26.3%		29.0%	24.5%	
Divorced/Separated/Widowed	44.6%	58.8%	41.9%		43.5%	45.0%	
*Total Energy Intake (kcal/day)*	1867.31 (1112.37)	1408.18 (811.99)	1954.52 (1141.74)	0.001*	3021.58 (1155.45)	1393.37 (655.03)	<0.001*
*Daily Total Fiber Intake (g/1000kcal)*	10.33 (4.11)	17.54 (3.89)	8.96 (2.35)	<0.001*	11.65 (4.67)	9.78 (3.75)	0.002*
*Daily Soluble Fiber Intake (g/1000kcal)*	3.32 (1.22)	5.36 (1.17)	2.93 (0.76)	<0.001*	3.69 (1.26)	3.16 (1.18)	0.004*
*Daily Insoluble Fiber Intake (g/1000kcal)*	7.01 (2.97)	12.17 (3.01)	6.03 (1.67)	<0.001*	7.97 (3.47)	6.61 (2.66)	0.002*
*Absolute Total Fiber Intake (g)*	18.19 (11.35)	24.52 (15.14)	16.98 (10.09)	0.008*	31.93 (10.98)	12.54 (4.78)	<0.001*
*MetS*				0.12			0.04*
Meets Criteria for MetS	59.2%	47.1%	61.5%		48.4%	63.6%	
Does Not Meet Criteria for MetS	40.8%	52.9%	38.5%		51.6%	36.4%	

Abbreviations: BMI, body mass index; MET, metabolic equivalent of task; SD, standard deviation; MetS, metabolic syndrome. Independent sample t-tests and chi-square tests were used to determine the p-values between groups.

* indicates statistical significance.

**Fig 1:**
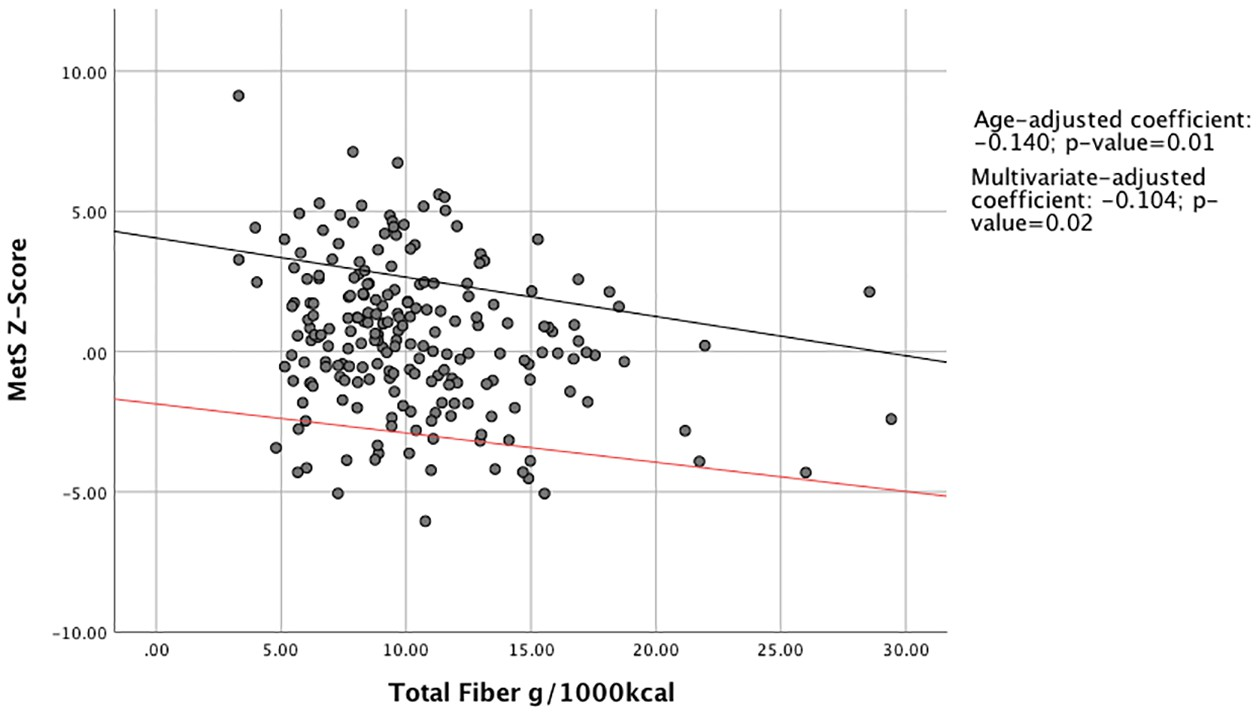
Association between total fiber intake and MetS Z-score. Abbreviations: MetS, Metabolic Syndrome. Linear regression model was used to predict association between daily fiber intake and MetS z-score. The black regression line indicates age-adjusted linear regression, for which the coefficient is -0.140. The red line indicates multivariate-adjusted regression, for which the coefficient is -0.104. Means were adjusted for age, BMI, smoking, education, marital status, total physical activity, and total energy intake. Both lines are statistically significant.

**Fig 2:**
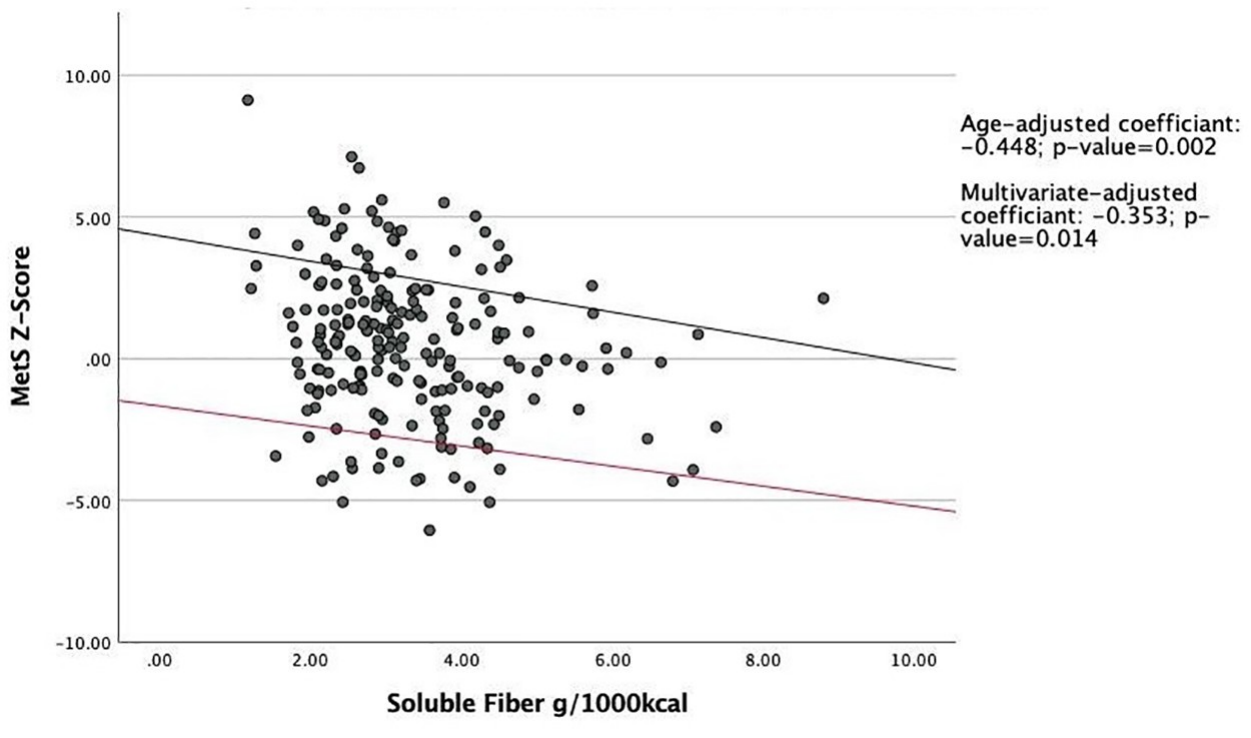
Association between soluble fiber intake and MetS Z-score. Abbreviations: MetS, Metabolic Syndrome. Linear regression model was used to predict association between soluble fiber intake and MetS z-score. The black regression line indicates age-adjusted linear regression, for which the coefficient is -0.448. The red line indicates multivariate-adjusted regression, for which the coefficient is -0.353. Means were adjusted for age, BMI, smoking, education, marital status, total physical activity, and total energy intake. Both lines are statistically significant.

**Fig 3:**
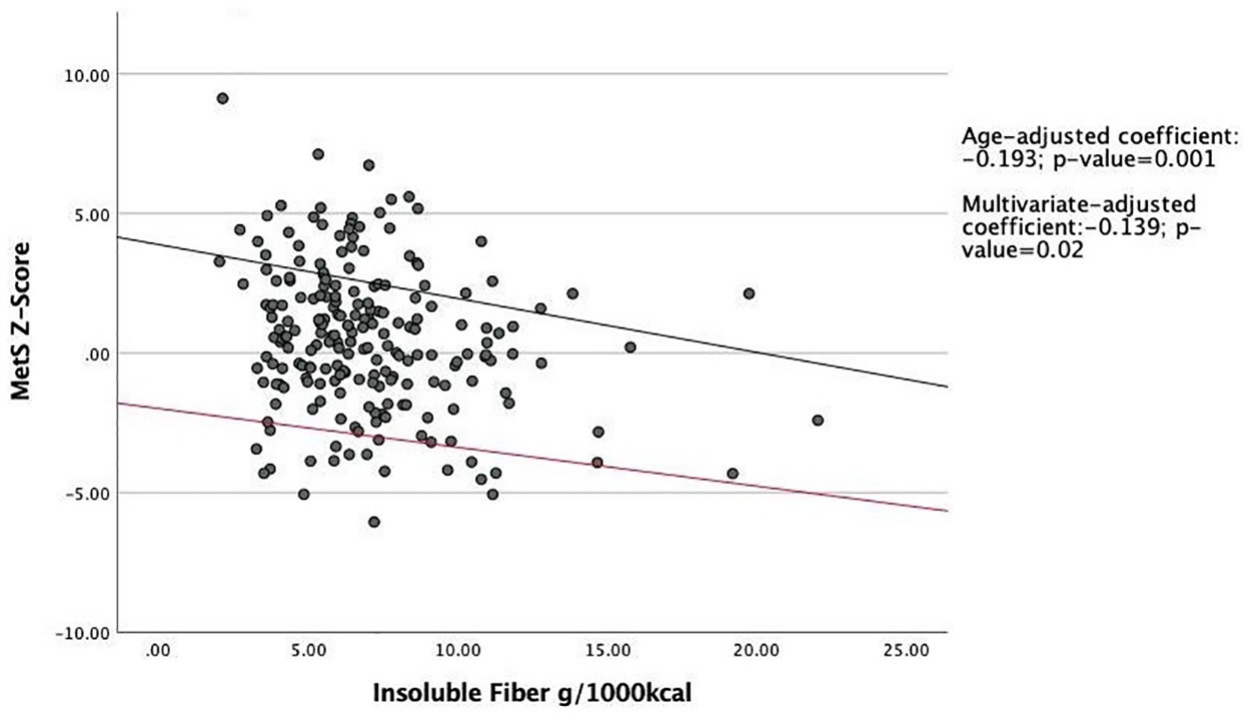
Association between insoluble fiber intake and MetS Z-score. Abbreviations: MetS, Metabolic Syndrome. Linear regression model was used to predict association between insoluble fiber intake and MetS z-score. The black regression line indicates age-adjusted linear regression, for which the coefficient is -0.193. The red line indicates multivariate-adjusted regression, for which the coefficient is -0.139. Means were adjusted for age, BMI, smoking, education, marital status, total physical activity, and total energy intake. Both lines are statistically significant.

The associations between the MetS z-score and total fiber, soluble fiber, and insoluble fiber were examined using age-adjusted as well as multivariate-adjusted (i.e. age, BMI, smoking, education, marital status, and total physical activity) linear regression models. Study participants with higher intakes of fiber per 1000 kcal/day had a statistically significant lower MetS z-score compared to participants who consumed less fiber. In age-adjusted and multivariable-adjusted linear regression models with MetS Z-score as the primary outcome, each unit increase in total fiber intake resulted in a decrease in the Z-score by 0.14 (P = 0.01) units and 0.10 (P = 0.02) units, respectively (Fig 1). Similar results were obtained for the associations of soluble and insoluble fiber with MetS Z-score (Figs 2 and 3).

The difference in means between individual MetS components among participants who met daily fiber intake recommendations compared to those who did not are presented in Table 2. Although mean levels of individual MetS components were not significantly different between those consuming at least 14 g per 1000kcal/day of fiber than those who did not, mean MetS z-score difference between the two groups was statistically significant. Mean z-scores were 1.41 units lower (P = 0.003) in those who met the daily fiber recommendations than those who did not. In analyses based on adherence to absolute fiber intake recommendations (21 g/day), higher mean HDL intake was associated with higher fiber intake. However, the z-score difference was not statistically significant (Table 2).

**Table 2:** Differences in MetS components by daily fiber recommendations per 1000kcal and daily total fiber recommendations.

	Fiber Intake ≥14g/1000 kcal per day (n = 34)	Fiber Intake <14 g/1000 kcal per day (n = 179)	Mean Difference	P-value	Fiber Intake ≥21 g/day (n = 62)	Fiber Intake <21 g/day (n = 151)	Mean Difference	P-Value
MetS Components	Mean (SD)	Mean (SD)			Mean (SD)	Mean (SD)		
Systolic Blood Pressure (mmHg)	132.74 (20.13)	131.80 (16.81)	0.93	0.76	132.92 (18.92)	131.56 (16.69)	1.36	0.60
Diastolic Blood Pressure (mmHg)	80.74 (11.66)	82.56 (10.76)	-1.83	0.37	82.31 (10.99)	82.26 (10.90)	0.05	0.98
Fasting Glucose (mg/dL)	99.18 (12.17)	103.57 (13.39)	-4.39	0.08	102.13 (13.80)	103.17 (13.09)	-1.04	0.60
Triglycerides (mg/dL)	94.57 (54.71)	116.84 (63.74)	-22.27	0.06	118.02 (62.32)	111.34 (63.10)	6.68	0.48
HDL Cholesterol (mg/dL)	67.35 (26.17)	58.82 (16.11)	8.54	0.07	64.94 (21.96)	58.23 (16.22)	6.71	0.03*
Waist Circumference (cm)	107.56 (15.71)	109.78 (13.69)	-2.22	0.40	108.92 (13.05)	109.64 (14.43)	-0.72	0.74
Mets Z-Score^#^	-0.67 (2.31)	0.74 (2.59)	-1.41	0.003*	0.27 (2.68)	0.62 (2.56)	0.35	0.38

Abbreviations: SD, standard deviation; HDL, high-density lipoprotein; CI, confidence interval; MetS, metabolic syndrome. Daily fiber recommendations are 14 g per 1000kcal/day. Means were tested by independent sample t-tests.

^#^ MetS z-score is a continuous score calculated based on ATPIII criteria and the standard deviations for each MetS component in the study sample

* indicates statistical significance.

Associations between the dichotomized MetS variables (yes/no) and adherence to daily fiber intake recommendations are presented in Table 3. In multivariate-adjusted analyses, participants not adherent to absolute fiber intake recommendations (21g/day or more) had a 4.24 (95% CI: 1.75, 10.30) times higher likelihood of having MetS as compared to those reporting intakes in the recommended range (P = 0.001). Of the MetS components, high fasting glucose and high triglycerides were associated with lower intake of fiber (P = 0.01 and P = 0.05, respectively). However, associations were not found when analyzing the adherence to daily fiber intake guidelines of 14g/1000kcal (Table 3).

**Table 3:** Associations of MetS and its components in those not adherent to fiber recommendations compared to those adherent to fiber recommendations.

	Adherence to 14g/1000kcal per day Recommendations		Adherence to 21g/day Recommendations	
	OR (95% CI)	p-value	OR (95% CI)	p-value
MetS	1.67 (0.76–3.67)	0.20	4.24 (1.75–10.30)	0.001*
High Systolic Blood Pressure	1.31 (0.60–2.89)	0.50	0.79 (0.35–1.79)	0.57
High Diastolic Blood Pressure	1.23 (0.55–2.73)	0.61	1.49 (0.65–3.40)	0.35
High Fasting Glucose	1.4 (0.63–3.10)	0.41	3.26 (1.34–7.93)	0.01*
High Triglycerides	1.66 (0.52–5.36)	0.39	3.02 (0.99–9.23)	0.05*
Low HDL	0.87 (0.38–1.97)	0.74	1.22 (0.50–2.99)	0.66
High Waist Circumference	n/a	n/a	n/a	n/a

Abbreviations: MetS, metabolic syndrome; OR, odds ratio; CI, confidence interval; HDL, high-density lipoprotein. Odds were tested by logistic regression test, and adjusted for age, BMI, smoking, education, marital status, total physical activity, and total energy intake.

* indicates statistical significance.

Waist circumference was not included in the analysis, because all participants had waist circumference ≥ 88cm as an inclusion criterion for the FIERCE study.

## Discussion

Postmenopausal African American women with obesity who consumed a higher amount of fiber had a better overall metabolic profile and were less likely to have MetS in our study. Even in a population with high prevalence of obesity, we observed variability in fiber intake corresponding to risk of MetS. Our findings highlight the need to focus beyond just weight reduction in these high-risk populations and investigate approaches to biologically modify and intercept chronic disease and cancer risk.

Despite this population consuming less than adequate amounts of dietary fiber, intake was comparable to the US reference population [20]. The age range of participants was between 45 years and 67 years and can be compared to those who participated in the National Health and Nutrition Examination Survey (NHANES) in 2015–2016. While mean energy-adjusted daily fiber intake among this study population was 10.33 g per 1000kcal/day, it was slightly higher than the reported mean in the national survey. Women in the 40–49 age range reported 8.5g per 1000kcal/day of fiber, while women in the 50–59 and 60–69 age ranges reported 9.2 g per 1000 kcal/day and 9.7g per 1000kcal/day, respectively [20]. Furthermore, in this population of postmenopausal African American women, fiber intake was higher than African Americans ages 20 years and older in the NHANES study, who had a mean intake of 7.3g per 1000kcal/day [20]. Other studies have also reported lower adherence with dietary fiber intake recommendations in urban African American populations that correlates with higher MetS prevalence [21].

Our study found that there are significant relationships between MetS, its components, and daily fiber when it was expressed in both absolute terms and by energy intake. Although women over 50 years old should be consuming at least 21 g/day (14 g per 1000kcal/day) based on IOM recommendations, other studies have found that risk reductions in diseases such as cardiovascular disease, stroke, and type 2 diabetes, were greatest when daily dietary fiber intake was even higher—between 25 and 29 grams [22]. Improved weight management was also significantly affected by an increase in fiber intake by ≥10–12 g/day [23]. These findings are important for informing future interventions to increase fiber intake in African American women who are metabolically unhealthy.

Higher intake of fiber in our study was associated with a better metabolic profile, especially when all MetS components were combined into a continuous z-score. The association showed linear benefit of increasing fiber intake (both soluble and insoluble) with decreasing MetS risk. A previous meta-analysis of cross-sectional studies found similar results of inverse relationships between daily fiber intake and MetS [24]. The meta-analysis also suggested that different types of fiber had varying effects on MetS prevalence [24]. However, our results suggested that daily total, soluble, and insoluble fiber intake, when expressed as g per 1000kcal, were all associated with lower MetS z-scores.

Other studies have suggested that varying levels of intake of soluble versus insoluble fiber might have different effects on participants’ metabolic profile [25, 26]. However, the findings from these studies have been contradictory. In a study among Spanish men and women, prevalence of MetS was analyzed based on levels of soluble and insoluble fiber [21]. The prevalence of MetS was lowest in participants who were in a higher quartile of insoluble fiber intake, while no significant association was found for soluble fiber [27]. This is in contrast with our study that found similar statistically significant associations for both soluble and insoluble fiber intake with MetS.

Individual MetS components, including fasting glucose and triglycerides, were found to have significant associations with fiber intake in our study. While participants who did not consume the recommended daily intake of fiber had higher odds of unhealthy levels of fasting glucose and triglycerides, they did have lower mean levels of HDL cholesterol. Previous studies have compared reported lifestyle behaviors, including fiber intake, in metabolically healthy and unhealthy participants. In a comparison of young women who were metabolically unhealthy and overweight/obese to healthy women, Camhi et al. reported statistically significantly higher fasting glucose and triglycerides, lower HDL cholesterol, and lower fiber intake [28]. Furthermore, their population of metabolically unhealthy women had lower levels of physical activity compared to metabolically healthy women, which may also contribute to a metabolically healthy profile [28]. Other studies in different populations (e.g., Chinese women) have reported associations of high dietary fiber with other MetS components, such as, increased HDL cholesterol [29]. We could not assess associations of fiber intake with abdominal obesity as all women in our study had abdominal obesity.

### Limitations of the study

Our study had several limitations. First, only baseline cross-sectional data was analyzed within this trial aimed at increasing exercise among metabolically unhealthy women–we did not have follow-up data, and dietary assessments were not conducted prior to MetS assessments. Second, due to the self-report nature of the FFQ, it is possible that there were inaccuracies with reporting–especially with the higher report of caloric intake. However, we used energy-adjusted fiber intake levels to account for this issue. Third, the study population only included women who were sedentary and metabolically unhealthy with obesity,. and the results may not be representative of all post-menopausal African American women. Lastly, the sample size, particularly for the group who adhered to daily fiber intake recommendations, was relatively small which could have led to some analyses being underpowered.

## Conclusions

In this cross-sectional analysis of baseline data from a clinical trial, study participants who consumed a higher amount of fiber had a better overall metabolic profile and were less likely to have MetS in our study of postmenopausal African American women with obesity. However, less than one-third of the study population met the daily recommendations for fiber intake. These findings might be important for designing interventions to reduce chronic disease and cancer risk in post-menopausal African American women. Given the alarming prevalence of obesity in the US population, approaches beyond weight reduction should be considered in metabolically unhealthy populations.

## Supporting information

S1 Dataset (XLSX)
